# Case Report: Hyper IgE, but Not the Usual Suspects–Kimura Disease in an Adolescent Female

**DOI:** 10.3389/fped.2021.674317

**Published:** 2021-07-20

**Authors:** Prasanna Venkatesh Ramachandran, C. Mary Healy, Elton M. Lambert, Deyanara Guerra, Choladda V. Curry, Tiphanie P. Vogel

**Affiliations:** ^1^Department of Pediatrics, Baylor College of Medicine, Houston, TX, United States; ^2^Pediatrician-Scientist Program, Baylor College of Medicine, Houston, TX, United States; ^3^Texas Children's Hospital, Houston, TX, United States; ^4^Division of Infectious Disease, Department of Pediatrics, Baylor College of Medicine, Houston, TX, United States; ^5^Division of Otolaryngology, Department of Pediatrics, Baylor College of Medicine, Houston, TX, United States; ^6^Department of Pathology and Immunology, Baylor College of Medicine, Houston, TX, United States; ^7^Division of Rheumatology, Department of Pediatrics, Baylor College of Medicine, Houston, TX, United States

**Keywords:** Kimura Disease, IgE, T helper 2, IgG4-related disease, angiolymphoid hyperplasia with eosinophilia, lymphocytic hypereosinophilic syndrome

## Abstract

Elevated immunoglobulin E (IgE) levels can be associated with infectious, allergic and inflammatory disorders, and rarely as a manifestation of an inborn error of immunity. Here we report the case of an adolescent female who presented with a gradually enlarging neck mass, lymphadenopathy, eosinophilia and highly elevated IgE levels. Laboratory and histopathologic evaluation revealed an unlikely diagnosis of Kimura Disease. We discuss the differential diagnosis of a neck mass with prominent eosinophils on histology, and review support for T-helper type 2 (Th2) cell activation and hyper-IgE in Kimura Disease.

## Introduction

Kimura disease (KD) is a rare, chronic inflammatory disorder of unknown etiology. It is characterized by the development of subcutaneous lymphoid masses, usually in the head and neck region, with associated regional lymphadenopathy (1, 2). Although solitary painless nodules are most common, KD patients with multiple nodules have been described (3). It occurs predominantly in young men of Asian descent, with a peak incidence in the second and third decade of life; presentation in childhood is rare (4, 5). Surgical removal of KD lesions may be curative, but recurrences are common and, in some cases, have been treated with corticosteroids and other immune modulators (3). A subset of KD patients also experiences kidney disease, typically nephrotic syndrome (3).

The pathophysiology of KD remains unclear. There is consideration that KD is a dysregulation of the immune system that results from an aberrant response following a normal stimulus, perhaps originally triggered by an infection or allergy (6–9). Diagnosis is confirmed by pathological analysis of the affected tissue, which typically displays dense inflammatory infiltrates including eosinophils and lymphoid follicles with germinal centers containing IgE (10). Laboratory findings characteristic of KD include peripheral eosinophilia and highly elevated levels of serum IgE (10). IgE production by B cells relies on isotype-switching, a process that requires coordinated interaction between B and T cells, as well as the secretion of soluble factors like interleukin-4 (IL-4), IL-5 and IL-13 by T-helper type 2 (Th2) cells (11, 12). Aberrant activation of signaling cascades may underlie the elevated IgE levels in KD.

Elevated IgE is seen in a broad spectrum of disorders and so has a large differential diagnosis. Allergic and respiratory diseases, such as atopic dermatitis and asthma, can be characterized by elevated IgE levels (13, 14). Certain parasitic and viral infections are also associated with elevated serum IgE (13, 14). A growing number of inborn errors of immunity can also present in rare patients with highly elevated IgE levels. This includes autosomal dominant hyper-IgE syndrome and related disorders, Wiskott-Aldrich syndrome and Immune dysregulation, Polyendocrinopathy, Enteropathy, X-linked (IPEX) syndrome (14, 15). Additionally, inflammatory diseases such as eosinophilic granulomatosis with polyangiitis, one of the anti-neutrophil cytoplasmic antibody (ANCA)-associated vasculitides, can have characteristic elevation of serum IgE (16, 17).

Here we present the case of a 14-year-old girl with a gradually enlarging mass of the left neck, peripheral eosinophilia and highly elevated IgE.

## Case Description

### History of Present Illness

A 14-year-old Filipino female was referred to evaluate and manage a growing left-sided neck mass.

She had initially developed neck swelling 10 months prior to presentation at our institution. Her previous exam and evaluation, including neck ultrasound and MRI, had noted lymphadenopathy in the left neck region, with one prominent lymph node in the left upper posterior cervical chain, and a second smaller post-auricular node. The etiology was initially thought to be infectious, secondary to a recent gingival infection, and she was treated with antibiotics without improvement.

Her left neck swelling gradually worsened over the course of several weeks and she was subsequently evaluated by multiple subspecialists. Laboratory evaluations were not revealing for an etiology. MRI of the chest/abdomen/pelvis was negative for additional lymph node or organ enlargement. Needle biopsy of the neck mass followed by flow cytometry was negative for signs of malignancy. Excision of the nodes was attempted and the smaller post-auricular node was successfully excised; however, the larger node was not able to be fully excised due to intra-operative bleeding.

Pathology on the tissue from the incisional biopsy showed no evidence of malignancy and cultures were negative. It did demonstrate reactive lymphadenopathy with follicular and interfollicular hyperplasia, increased eosinophils, focal vascular proliferation and IgG4+ cells. Serum IgG4 levels were within normal limits.

A working diagnosis of IgG4-related disease (IgG4-RD) was made, and the patient was started on 60 mg prednisone daily with near complete resolution of the neck mass over the course of several months. However, with taper down to 15 mg prednisone daily there was recrudescence, and the mass grew back to pre-treatment size when steroids were discontinued. She developed some pain with neck movement due to the mass, but there was no difficulty with breathing or swallowing. She had experienced side effects from the high-dose steroids including weight gain and irritability.

The family moved and presented to our institution to continue care.

### Review of Systems

Otherwise unremarkable including that the patient and mother denied fevers, chills, night sweats, rashes, arthralgias, myalgias, hematuria, edema, frothy urine, hearing loss, sinusitis, epistaxis, cough, shortness of breath, hemoptysis.

### Past Medical History

The patient has intellectual disability and global developmental delay. Her history was also notable for Marfanoid features on exam, including increased arm span, positive thumb and wrist signs, mild kyphosis, pectus excavatum and myopia. Family declined evaluated by genetics, echocardiography was unremarkable. She had had bilateral adenoidectomy and tonsillectomy at the age of 12 years. There was no history of recurrent or severe infections.

### Social and Family History

The patient lives with her biologic parents and two younger sisters who are all healthy. The patient's paternal grandfather died of an unknown cancer, and her mother's cousin was diagnosed with systemic lupus erythematosus. The family denied any known unusual exposures to animals, insects or toxins. The patient was born in the Philippines and moved to the United States when she was 6 years old.

### Physical Examination

**Figure 1:**
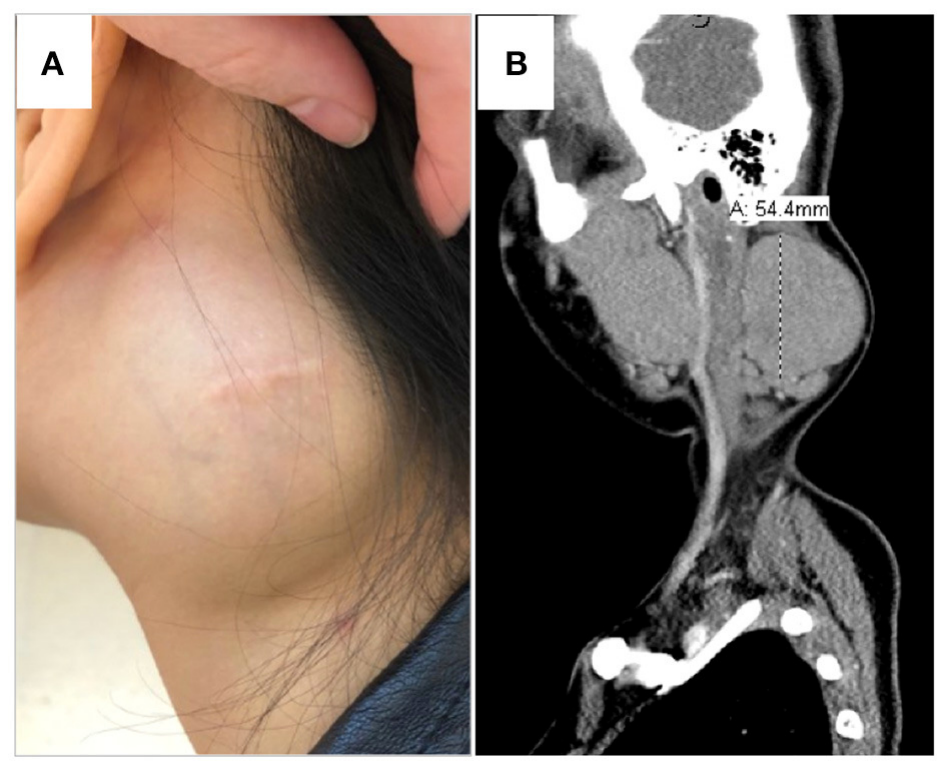
Left neck mass. **(A)** Physical exam displayed a prominent, firm left neck mass, roughly 5 × 5 cm, which was immobile, non-tender, non-fluctuant and marked by a healed surgical scar. She had an additional small, healed, post-auricular surgical scar. **(B)** CT scan demonstrated a 5.4 × 3.7 × 6.3 cm, well-delineated, non-calcified, homogenous, non-necrotic nodal mass.

The patient was afebrile and vital signs were unremarkable. She was a well-developed, well-nourished, female teenager in no apparent distress at the 88th percentile for both height and weight. There was a prominent, firm, left neck mass (Figure 1A), roughly 5 × 5 cm, which was immobile, non-tender, non-fluctuant and marked by a healed surgical scar. She had an additional small, healed surgical scar behind the left ear. She had full range of motion of all joints including her neck, but endorsed mild pain with lateral rotation. The remainder of her examination was unremarkable aside from the previously noted mild dysmorphisms.

### Laboratory Evaluation, Imaging, and Pathology Findings

Laboratory evaluation conducted at our institution is shown in Table 1 and Supplementary Table 1. Extensive evaluation for infectious etiology accounting for all potential exposures was unremarkable (Supplementary Table 2). C reactive protein was unremarkable and the highest erythrocyte sedimentation rate was 23 mm/h (normal <20 mm/h). Results were most notable for the presence of peripheral eosinophilia (2.9 K/uL) and highly elevated serum IgE (39,232 kU/L). Computed tomography (CT) imaging of the neck revealed left cervical chain lymphadenopathy, with a nodal mass measuring 5.4 × 3.7 × 6.3 cm (Figure 1B), and additional asymmetric nodes <1 cm scattered along the upper and lower left neck. No calcification or fluid collection was observed. Creatinine was within normal limits and there was no hematuria or proteinuria.

**Table 1:** Laboratory evaluation.

**Test, units**	**Value**	**Reference range**
WBC, × 10^3^/uL	10.5	4.5–13.5
Hemoglobin, g/dL	13.8	12.0–16.0
Platelets, × 10^3^/uL	191	150–450
Absolute neutrophils, cells/uL	4,770	1,800–8,000
Absolute lymphocytes, cells/uL	2,127	1,500–5,000
Absolute eosinophils, cells/uL	**2,955**	**100–300**
ESR, mm/hr	16–23	0–20
C reactive protein, mg/dL	0.6	<1.0
IgE, kU/L	**39,232**	**≤114**
IgA, mg/dL	171.0	66–295
IgM, mg/dL	159.0	40–80
IgG, mg/dL	1,600	641–1,353
IgG subclass 4, mg/dL	107.5	11–157

*WBC, white blood cells; ESR, erythrocyte sedimentation rate; Ig, immunoglobulin. Salient features of Kimura Disease are in bold*.

**Figure 2:**
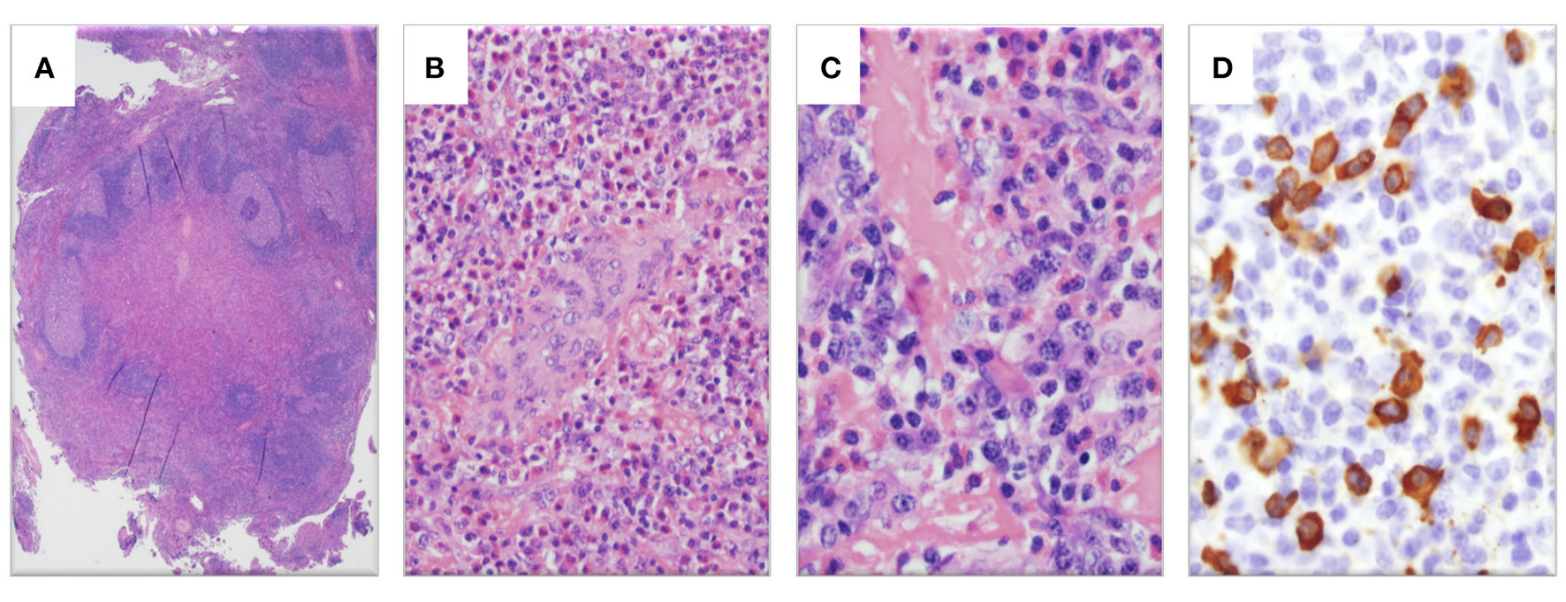
Histopathologic examination of left neck mass. **(A)** Hematoxylin and eosin (H&E)-stain showing lymphoid tissue with marked interfollicular expansion and reactive follicles (1.25×). **(B)** H&E-stain showing prominent eosinophilia and plasmacytosis together with plump endothelial cells (20×). **(C)** H&E-stain revealing a fibrotic process with elevated plasma cells and eosinophils (40×). **(D)** Abundant IgG4-stained cells (>60 per high powered field, 40×).

Pathologic re-evaluation of her biopsy (Figures 2A–D) confirmed reactive lymphoid hyperplasia with dense tissue eosinophilia including eosinophilic microabscesses, focal vascular proliferation, focal sclerosis and prominent plasmacytosis with increased IgG+ cells (>60 per high powered field, IgG4:IgG ratio 40–50%).

## Discussion

The patient is a 14-year-old female with a large left-sided neck mass, who had near-complete response to corticosteroids but recrudescence with taper. An initial diagnosis of IgG4-RD was made, after exclusion of infection and malignancy, based on biopsy findings of IgG4+ cells. Our additional evaluation noted peripheral eosinophilia and highly elevated serum IgE. Combined with reevaluation of the biopsy results, this led to revision of her diagnosis to Kimura Disease.

In this case, imaging did not reveal involvement of other anatomic locations and so lymphoproliferative disorders were less likely. Solitary neck masses have a broad differential diagnosis. In this patient, malignancy, such as lymphoma or metastatic disease, and infection, such as scrofula or parasitic lymphadenitis, were considered unlikely based on prior evaluation and her treatment response. Pathology was not consistent with a histiocytic disorder or unicentric Castleman Disease. Instead, based on histopathology the differential diagnosis included IgG4-RD, angiolymphoid hyperplasia with eosinophilia (ALHE) and Kimura Disease (Table 2). As ALHE and KD are typically approached with surgical resection as first-line therapy, but immune modulation is first line treatment for IgG4-RD, correct diagnosis is important for therapeutic recommendations.

**Table 2:** Disease comparison (18–20).

	KD^*^	**ALHE**	**IgG4-RD**	**L-HES**
**Demographics**				
Age	Young	Middle-age	Older	Middle-age
Sex	Male	Female	Male	Equal
Race	Asian	Equal	Equal	Equal
**Lesions**				
Location	Head and neck	Head and neck	Variable	Variable
Lymph node involvement	Yes	Rare	Yes	Rare
Number	Single or multiple	Multiple	Single or multiple	Multiple
Size (>2 cm)	Yes	No	Variable	No
Discoloration	Rare	Yes	Variable	Rare
Pruritus	Rare	Yes	Variable	Yes
Prior trauma	Rare	Yes	Variable	Rare
**Laboratory values**				
IgE elevated	Yes	Rare	Yes	Yes
Eosinophila	Yes	Rare	Yes	Yes
**Histology**				
Site	Subcutaneous tissue	Dermis	Variable	Variable, including internal organs
Demarcation	Poor	Good	Good	Poor
Vascular proliferation	Yes	Yes	No	No
Lymphoid follicles	Yes	Rare	Yes	No
Eosinophilic infiltrate	Dense	Variable	Variable	Dense
Histiocytoid endothelial cells	Rare	Yes	No	No
Fibrosis	Yes	Rare	Storiform	No
Plasma cells	Absent or sparse	Absent or sparse	Dense	Sparse
IgG4/IgG ratio	Not diagnostic	Not diagnostic	>40%	Not diagnostic

* *KD, Kimura Disease; ALHE, angiolymphoid hyperplasia with eosinophilia; IgG4-RD, Immunoglobulin G4-related disease; L-HES, lymphocytic hypereosinophilic syndrome*.

### IgG4-Related Disease

IgG4-RD is a rare, chronic immune-mediated disorder that can cause fibroinflammatory lesions in nearly any organ (21). It is a predominately adult-onset condition that generally presents as multiorgan disease and may be confused early in the course for malignancy, infection, or other immune-mediated conditions. Typical pathologic findings include a lymphoplasmacytic infiltrate, storiform fibrosis, obliterative phlebitis, and IgG4+ plasma cell infiltrates (22). Serum IgG4 levels may be normal, and this finding is not central to the diagnosis (22). Characteristic radiologic findings, such as a sausage-shaped pancreas or periaortitis of the lower aorta, are strongly associated with IgG4-RD. However, no single clinical, serologic, histologic or radiographic finding is considered definitive for the diagnosis (23). An international consortium of physicians from the American College of Rheumatology (ACR) and the European League Against Rheumatism (EULAR) jointly issued newly updated classification criteria for IgG4-RD in 2019 (24). The diagnosis requires clinical correlation with serologic and histopathologic data. A 3-step classification process was developed comprising entry, exclusion and inclusion criteria. These criteria were validated in a large cohort of patients with robust sensitivity and specificity (24). Based on these classification criteria, peripheral eosinophilia > 3,000 cells/uL is an exclusion for IgG4-RD that our patient was just shy of fulfilling. However, her pathologic findings did not meet enough criteria to result in a diagnosis of IgG4-RD, as cells staining positive for IgG4 is insufficient to diagnosis IgG4-RD without other characteristic pathologic findings and organ involvement.

### Angiolymphoid Hyperplasia With Eosinophilia

ALHE shares sufficient clinical and histopathologic features with KD that investigators in the past wondered if these entities were part of the same disease spectrum, although they are now considered by most to be distinct diseases (18, 25). While the lesions of ALHE are also generally located in the head and neck region, they tend to be smaller and are more often found in multiples (18). ALHE is also more common in females and the lesions are often described as discolored and pruritic (18). Although the peak incidence of ALHE is in the second to fourth decade of life, ALHE has been described in all age groups (25). Unlike KD, ALHE is not increased in Asian patients (18). While both ALHE and KD demonstrate vascular proliferation on histology, the vessels in ALHE are abnormal with characteristically plump, histiocytoid endothelial cells with cytoplasmic vacuoles (18, 25). ALHE demonstrates a lymphocytic infiltrate but is less likely to have dense eosinophils and typically does not have follicles (18). Peripheral eosinophilia and elevated IgE are uncommon in ALHE (18). Therefore, the laboratory findings of eosinophilia and elevated IgE combined with the histologic findings of reactive follicles and dense tissue eosinophilia including microabscesses favored a diagnosis of KD.

### Lymphocytic Hypereosinophilic Syndrome

The hypereosinophilic syndromes (HES) are a heterogeneous group of rare disorders that should be considered when peripheral eosinophilia > 1.5 × 10^9^/L cells is present. A distinct subset of HES patients manifest with a T-cell-mediated polyclonal expansion and have what is termed lymphocytic HES (L-HES) (19, 20). L-HES affects females and males equally, and most commonly presents in between age 20 and 50 years, although it can develop in children. L-HES is most commonly characterized by cutaneous manifestations (pruritus, eczema, urticaria, angioedema) and lymphadenopathy, but symptoms can be present as the result of eosinophils in other organ systems including respiratory, cardiovascular and musculoskeletal. Serum IgE levels are often increased. Although short-term prognosis in L-HES is excellent, there is a significant predisposition to T-cell lymphoma in this disorder.

### Elevated IgE With Th2 Activation in KD

The pathogenesis of KD is unknown. Various investigators have suggested a disorder of immune dysregulation following an allergic response, consistent with the presence of eosinophilia and increased IgE (6, 9, 17, 26). The suggestion that KD patients can commonly have eczema further supports this (27). Rare immunophenotyping data is available from KD patients. Katagiri et al. reported upregulated transcript levels of IL-4, IL-5 and IL-13, but not interferon γ, in peripheral blood cells from a patient with KD (9). Hosoki et al. demonstrated increased IL-5 expressing cells in a case of pediatric KD, as well as increased IL-10 expressing cells (6). Elevated percentages of peripheral blood Th2 cells (CD4+ T cells expressing IL-4) correlated with IgE levels in KD patients in a study by Ohta et al. who also demonstrated elevated tissue IL-5 (28). Th2 cytokines not only induce IgE synthesis, but also promote infiltration of eosinophils into the tissue, both hallmarks of KD pathogenesis. Other investigators have suggested that allergic cytokines produced by basophils and mast cells also contribute to the elevation of IgE in KD (7, 8). While support for aberrant allergic disease has been shown, additional studies will be necessary to fully unravel the etiology of KD.

### Patient Outcome

**Figure 3:**
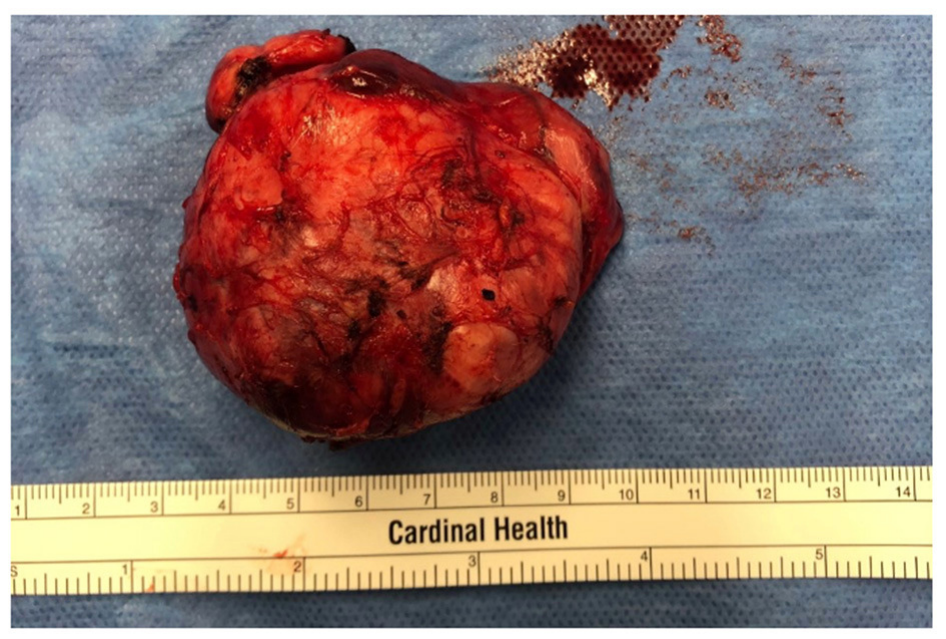
Gross surgical specimen. The nodal mass was completely excised.

Standards for treatment of KD have not been established. Options include surgical excision, local radiation, combined excision and radiation, systemic corticosteroids, or, rarely, other immune modulators (5, 29). Our patient had known recrudescence of the mass following corticosteroid taper. She also had no evidence of disease outside the left neck mass. The decision was made to excise the mass for an attempt at definitive therapy (Figure 3), and the patient tolerated the procedure well. Subsequent evaluations revealed normalized inflammatory markers and peripheral eosinophils, and downtrending IgE (nadir 4,448 kU/L). One year after surgery, the patient noted the recurrence of her left sided neck mass and is considering immune modulation with cyclosporine. The patient will continue to be closely followed for the development of renal disease (26).

## Data Availability Statement

The original contributions generated for the study are included in the article/supplementary material, further inquiries can be directed to the corresponding author/s.

## Ethics Statement

Ethical review and approval was not required for this study on human participants in accordance with the local legislation and institutional requirements. Written informed consent to participate in this study was provided by the participants' legal guardian/next of kin. Written informed consent was obtained from the minor(s)' legal guardian/next of kin for the publication of any potentially identifiable images or data included in this article.

## Author Contributions

PR and TV: conception or design of the work. PR, CH, EL, DG, CC, and TV: data collection and critical revision of the article. PR, CC, and TV: data analysis and interpretation. PR: drafting the article. All authors contributed to the article and approved the submitted version.

## Conflict of Interest

The authors declare that the research was conducted in the absence of any commercial or financial relationships that could be construed as a potential conflict of interest.
