# Supplementary material for: Case Report: Hyper IgE, but Not the Usual Suspects–Kimura Disease in an Adolescent Female
Source: Front Pediatr. 2021 Jul 20;9:674317. doi: 10.3389/fped.2021.674317 (PMC8329340; doi:10.3389/fped.2021.674317)
Supplement: Supplementary file 1 [file Table_1.doc]

| **Test, units** | **Value** | **Reference range** |
| --- | --- | --- |
| WBC, x10^3^/uL | 10.5 | 4.5 - 13.5 |
| Hemoglobin, g/dL | 13.8 | 12.0 - 16.0 |
| Platelets, x10^3^/uL | 191 | 150 - 450 |
| Neutrophils, % | 45.6 | 33 - 76 |
| Lymphocytes, % | 20.3 | 15 - 55 |
| Monocytes, % | 4.2 | 0 - 4 |
| Eosinophils, % | 28.2 | 0 - 3 |
| Basophils, % | 1.4 | 0 - 1 |
| Absolute Neutrophils, cells/uL | 4,770 | 1,800 - 8,000 |
| Absolute Lymphocytes, cells/uL | 2,127 | 1,500 - 5,000 |
| Absolute Eosinophils, cells/uL | 2,955 | 100 - 300 |
| Creatinine, mg/dL | 0.83 | 0.50 - 0.80 |
| AST, U/L | 13 | 10 - 30 |
| ALT, U/L | 6 | 10 - 35 |
| Alkaline Phosphatase, U/L | 106 | 62 - 209 |
| LDH, U/L | 457 | 390 - 580 |
| Albumin, g/dL | 4.7 | 3.5 - 5.2 |
| Total Protein, g/dL | 7.5 | 6.8 - 8.5 |
| Ferritin, ng/mL | 18 | 10 - 70 |
| Thyroxine (T4), ug/dL | 7.8 | 4.5 - 10.0 |
| TSH uIU/mL | 1.927 | 0.5 - 3.4 |
| ESR, mm/hr | 16 - 23 | 0 - 20 |
| C Reactive Protein, mg/dL | 0.6 | <1.0 |
| IgE, kU/L | 39,232 | <=114 |
| IgA, mg/dL | 171.0 | 66 - 295 |
| IgM, mg/dL | 159.0 | 40 - 80 |
| IgG, mg/dL | 1,600 | 641 - 1,353 |
| IgG Subclass 1, mg/dL | 545 | 315 - 855 |
| IgG Subclass 2, mg/dL | 385 | 64 - 495 |
| IgG Subclass 3, mg/dL | 70 | 23 - 198 |
| IgG Subclass 4, mg/dL | 107.5 | 11 - 157 |
| Interferon gamma, pg/mL | <5 | <=5 |
| IL-1beta, pg/mL | <5 | <=36 |
| IL-2, pg/mL | <5 | <=12 |
| IL-2 Receptor, Soluble, pg/mL | 474 | <=1,033 |
| IL-4, pg/mL | <5 | <=5 |
| IL-5, pg/mL | <5 | <=5 |
| IL-6, pg/mL | <5 | <=5 |
| IL-8, pg/mL | <5 | <=5 |
| IL-10, pg/mL | <5 | <=18 |
| IL-12, pg/mL | <5 | <=6 |
| IL-13, pg/mL | <5 | <=5 |
| IL-17, pg/mL | <5 | <=13 |

**Supplemental Table 1**: Laboratory Evaluation

WBC, white blood cell; AST, aspartate aminotransferase; ALT, alanine amino- transferase; LDH, lactate dehydrogenase; TSH, thyroid stimulating hormone; ESR, erythrocyte sedimentation rate; Ig, immunoglobulin; IL, interleukin
