# Supplementary material for: Case Report: Hyper IgE, but Not the Usual Suspects–Kimura Disease in an Adolescent Female
Source: Front Pediatr. 2021 Jul 20;9:674317. doi: 10.3389/fped.2021.674317 (PMC8329340; doi:10.3389/fped.2021.674317)
Supplement: Supplementary file 2 [file Table_2.doc]

**Supplemental Table 2**: Infectious Evaluation

| **Test, units** | **Value** | **Reference range** |
| --- | --- | --- |
| Aspergillus, titers | <1:8 |  |
| *Bartonella henselae* IgG | <1:64 |  |
| *Bartonella henselae* IgM | <1:16 |  |
| *Bartonella quintana* IgG | <1:64 |  |
| *Bartonella quintana* IgM | <1:16 |  |
| Blastomyces, IV | 0.3 | <=0.9 |
| Coccidioides, titers | <1:2 | <1:2 |
| Hepatitis B Core Antibody | negative |  |
| Hepatitis B Surface Antigen | negative |  |
| Hepatitis A IgG | positive |  |
| Hepatitis A IgM | negative |  |
| Hepatitis C Antibody | negative |  |
| Histoplasma, Mycelial Phase | <1:8 |  |
| Histoplasma, Yeast Phase | <1:8 |  |
| Histoplasma Antigen | none detected |  |
| HIV Antibody | nonreactive |  |
| HIV1 RNA | not detected |  |
| Ova and Parasites | None seen |  |
| *Strongyloides* IgG, IV | 0.7 | <=0.9 |
| *Toxoplasma* IgG, IU/mL | 5.4 | <7.2 |
| *Toxoplasma* IgM, AU/mL | 5.2 | <8.0 |
| *Treponema* Antibody | negative |  |
| Tuberculosis (TSPOT) | negative |  |
| *Toxocara* Antibody | negative |  |
| *Trichinella* Antibody | negative |  |
